# Evaluation of perivascular fat density and residual false lumen formation following TEVAR in Stanford type B aortic dissection

**DOI:** 10.3389/fcvm.2025.1633817

**Published:** 2025-06-13

**Authors:** Xin He, Yubin Zhong, Hua Cao, Zhangbo Cheng

**Affiliations:** ^1^ Shengli Clinical Medical College, Fujian Medical University, Fuzhou, China; ^2^ Department of Cardiovascular Surgery, Fuzhou University Affiliated Provincial Hospital, Fuzhou, China

**Keywords:** perivascular adipose tissue, thoracic endovascular aortic repair, Stanford type b aortic dissection, residual false lumen, Hounsfield unit, biomarker prediction

## Abstract

**Objective:**

This study aims to investigate the role of perivascular adipose tissue (PVAT) attenuation in predicting residual false lumen formation following thoracic endovascular aortic repair (TEVAR) in patients with Stanford Type B aortic dissection (TBAD). The focus is on the association between PVAT attenuation (HU_Δ_and HU_ratio_) and postoperative outcomes, particularly the development of residual false lumen.

**Methods:**

A retrospective analysis was conducted on 132 patients who underwent TEVAR for TBAD at Fujian Provincial Hospital between 2016 and 2024. Patients were classified into two groups based on postoperative imaging findings: those with residual false lumen and those with completely closed false lumen. Data collected included demographic, biochemical, and imaging parameters. PVAT was assessed using computed tomography angiography (CTA), with the TotalSegmenter deep learning model used for automatic segmentation. Two indicators-Hounsfield unit difference (HU_Δ_) and Hounsfield unit ratio (HU_ratio_)-were calculated.

**Results:**

Patients with residual false lumen showed significantly higher HU*_Δ_* (8.75 ± 3.29 vs. 5.16 ± 2.84, *P* < 0.001) and lower HU_ratio_ (0.73 ± 0.13 vs. 0.85 ± 0.11, *P* < 0.001) compared to those with closed false lumen. Multivariate logistic regression identified HU*_Δ_*and HU_ratio_ as independent predictors of residual false lumen formation after TEVAR. ROC curve analysis revealed optimal cut-off values for predicting residual false lumen: HU_Δ_ > 7.170 (sensitivity 0.895, specificity 0.762) and HU_ratio_ ≤ 0.790 (sensitivity 0.947, specificity 0.667).

**Conclusions:**

PVAT attenuation, reflected by HU_Δ_ and HU_ratio_, serves as a significant, non-invasive imaging biomarker for predicting residual false lumen formation after TEVAR in TBAD patients. These findings suggest that preoperative evaluation of PVAT characteristics can help identify high-risk patients and guide postoperative management strategies. Further prospective studies are needed to validate these findings and explore the potential of PVAT modulation in improving long-term outcomes following TEVAR.

## Introduction

Aortic dissection (AD) is a life-threatening vascular disorder whose incidence has risen steadily in recent years (1, 2). Although the pathogenesis of AD is still not fully clarified, it is known to correlate closely with hypertension, hyperlipidaemia and inherited connective-tissue disorders such as Marfan syndrome (3, 4). Clinically, AD most often presents with sudden-onset chest or back pain described as a tearing sensation (5).Today, computed-tomography angiography (CTA) of the thorax and abdomen remains the diagnostic gold standard for AD because it clearly delineates intimal tears and the true-and false-lumen channels. According to the extent of intimal-tear involvement, AD is classified into Stanford Type A aortic dissection (TAAD), Stanford Type B aortic dissection (TBAD) and “non-A non-B” variants (6, 7). This anatomical distinction directs therapy: open surgical replacement is generally required for TAAD, whereas thoracic endovascular aortic repair (TEVAR) has become the first-line treatment for TBAD (8–10).

Despite the advantages of TEVAR, a frequent and clinically important complication is the persistence of a residual false lumen around the implanted stent graft. Such a persistent channel promotes late aortic aneurysm formation, raises rupture risk and can ultimately prove fatal (11–13). Elucidating the mechanisms that drive residual false-lumen formation after TEVAR, and intervening early, are therefore critical to improving long-term prognosis and quality of life for these patients.

Emerging evidence highlights the pathophysiological importance of perivascular adipose tissue (PVAT) in a spectrum of vascular diseases, including coronary artery disease and aortic aneurysms (14–16). Situated immediately adjacent to the vessel wall, PVAT mirrors local inflammatory and atheromatous activity and thus offers a promising, non-invasive window into vascular health. In abdominal aortic aneurysm progression, for instance, PVAT that lies closest to the wall displays larger density shifts than more distant adipose tissue (17, 18). Nonetheless, the influence of PVAT characteristics on vascular remodelling and clinical outcomes after TEVAR remains largely unexplored.

Accordingly, the present study investigates whether PVAT attenuation indices are associated with the development of a residual false lumen following TEVAR for TBAD. By clarifying this relationship, we aim to provide an early-warning biomarker that can guide surveillance intensity and inform timely adjunctive interventions in high-risk patients.

## Material and methods

### Patients

This study adhered to the ethical guidelines outlined in the Declaration of Helsinki and was approved by the Ethics Committee of Fujian Provincial Hospital (approval number K2025-02-111). Written informed consent was obtained from all participants.

### Patient selection and characteristics

We performed a retrospective analysis of patients with type B aortic dissection (TBAD) who underwent thoracic endovascular aortic repair (TEVAR) at the Department of Cardiovascular Surgery, Fujian Provincial Hospital, from January 1, 2016, to June 2024. The inclusion criteria were: (1) patients aged over 18 years with a diagnosis of TBAD; (2) presence of multiple ruptures between the true and false lumens of the dissection; (3) failure of the TEVAR stent to seal the distal rupture intraoperatively; (4) availability of comprehensive baseline clinical data, including age, gender, body mass index (BMI), smoking and alcohol history, diabetes, and hypertension; (5) laboratory data, including white blood cell count, neutrophil count, lymphocyte count, low-density lipoprotein (LDL), high-density lipoprotein (HDL), triglycerides, prothrombin time, activated partial thromboplastin time, and D-dimer levels; and (6) detailed surgical data, such as the time from diagnosis to surgery, stent oversizing rate, stent length/tear length, membrane material used for the stent(expanded polytetrafluoroethylene,ePTFE or Dacron).

**Figure 1:**
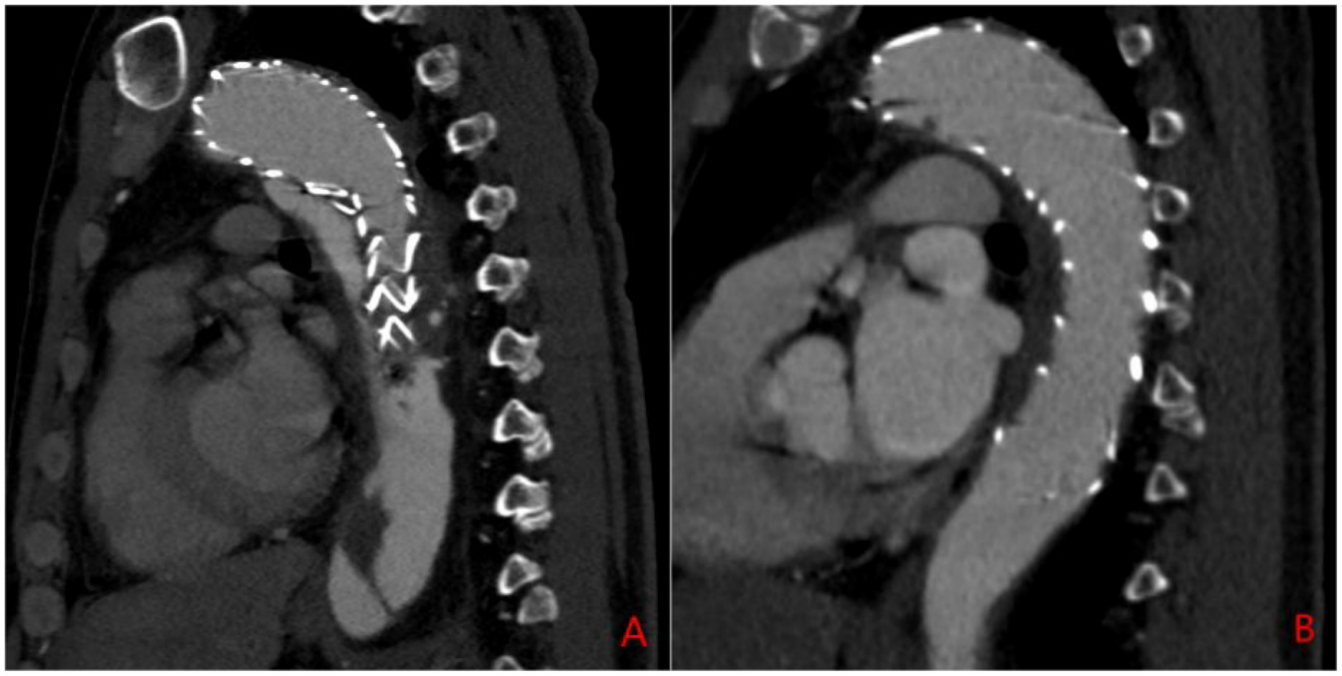
Sagittal CTA of the aorta post-TEVAR surgery. **(A)** Residual false lumen around the stent following TEVAR; **(B)** closure of the false lumen around the stent after TEVAR.

Exclusion criteria included: (1) the presence of aortic ulcers or hematomas; (2) incomplete baseline or imaging data; (3) poor-quality CTA imaging data; and (4) patients undergoing immunotherapy (e.g., chemotherapy or radiotherapy). Patients were then classified into two groups: Group 1 (residual false lumen group) and Group 2 (closed false lumen group) based on the presence or absence of a residual false lumen after TEVAR. The residual false lumen around the stent post-TEVAR is shown in Figure 1.

**Figure 2:**
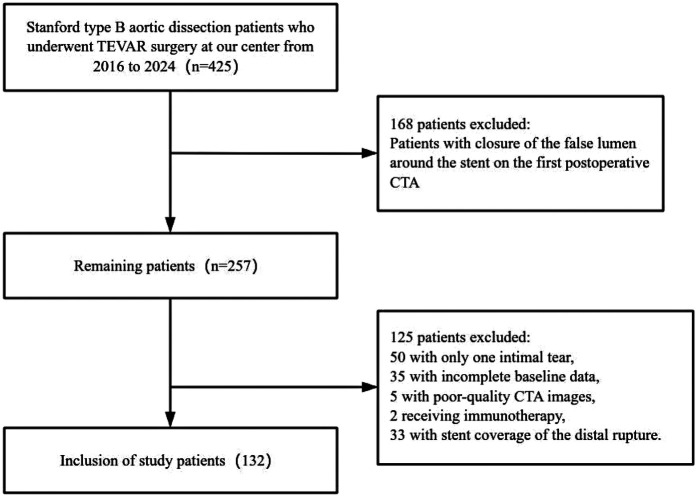
Flowchart of patient screening and inclusion criteria.

Clinical and laboratory variables were collected as follows: demographic data (age, gender, BMI, smoking and drinking history, diabetes, and hypertension), and blood biochemistry data including white blood cell count, neutrophil count, lymphocyte count, lipid profile (LDL, HDL, triglycerides), and coagulation-related markers such as prothrombin time, activated partial thromboplastin time, and D-dimer. Blood biochemistry data were collected postoperatively, with the first measurements taken on the day after TEVAR surgery. The lipid profile was measured on admission.The patient selection flowchart is shown in Figure 2.

### Image acquisition

CTA examinations were performed on a 64-detector row CT system (Brilliance 64; Philips Healthcare, Best, NL). Acquisition settings were identical for every patient: detector collimation 64 × 0.625 mm, pitch 0.90, rotation time 0.60s, tube voltage 120 kVp, and automated tube-current modulation targeting 300 mAs. Images were reconstructed with a medium-soft tissue convolution kernel B and iDose^4 iterative reconstruction (level 3), matrix 512 × 512, field-of-view 350 mm. Axial datasets were generated at 0.625-mm slice thickness with 0.3-mm increments. Daily phantom calibration was performed, and the same arterial-phase bolus-tracking protocol (trigger 120 HU, ROI at the descending thoracic aorta) was applied to all scans.

### Image annotation and AI automation

**Figure 3:**
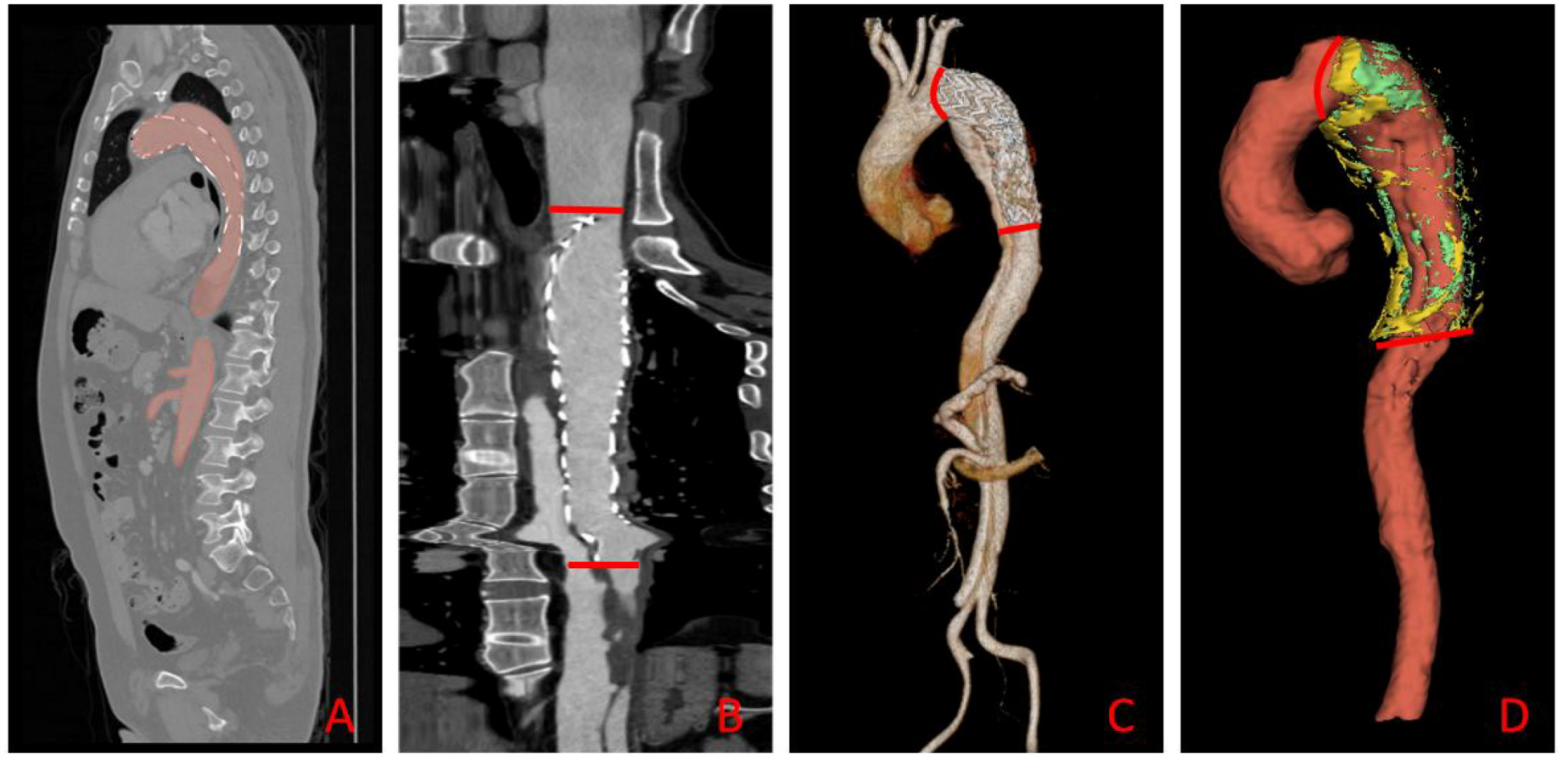
Automated aortic segmentation, range selection, and 3-D visualization workflow. **(A)** Sagittal CT slice showing the automatically segmented aortic mask (red overlay); **(B)** straightened (centerline-reformatted) aorta; the segment between the two red bars defines the quantitative analysis range. **(C)** Three-dimensional surface rendering of the aorta, with the same proximal and distal limits (red bars) demarcating the analysis segment. **(D)** Composite 3-D rendering of the selected aortic segment and its surrounding PVAT: the aorta is displayed in red, HU close voxels in green, and HU distance voxels in yellow.

To improve segmentation efficiency and standardize post-operative analysis, we used the open-source, pre-trained TotalSegmenter model built on the nnU-Net framework (19, 20). The model automatically delineated the aorta—from the right coronary ostium to the iliac bifurcation—and perivascular adipose tissue (PVAT) on contrast-enhanced CT, as shown in Figure 3. Although TotalSegmenter has not been externally validated for PVAT segmentation in this specific dataset, it has been widely validated across multiple other datasets. To ensure the accuracy of the segmentation, all automatic masks were independently reviewed by two radiologists. Any discrepancies were resolved by a senior cardiovascular radiologist. Additionally, manual corrections were made around the endovascular stent grafts to exclude metal-induced artefacts, ensuring the integrity of the segmentation results.

### PVAT segmentation workflow

Starting from the outer aortic adventitia generated by TotalSegmentator, we applied two concentric 3-D dilations (radial offsets 2–5 mm and 10–12 mm) to define the proximal (HU_close_) and distal (HU_distant_) PVAT rings, respectively. Voxels with attenuation between −180 HU and −30 HU were retained to identify adipose tissue (21). This workflow was executed by a single Python batch script, ensuring identical radial offsets and density thresholds across all patients.

**Figure 4:**
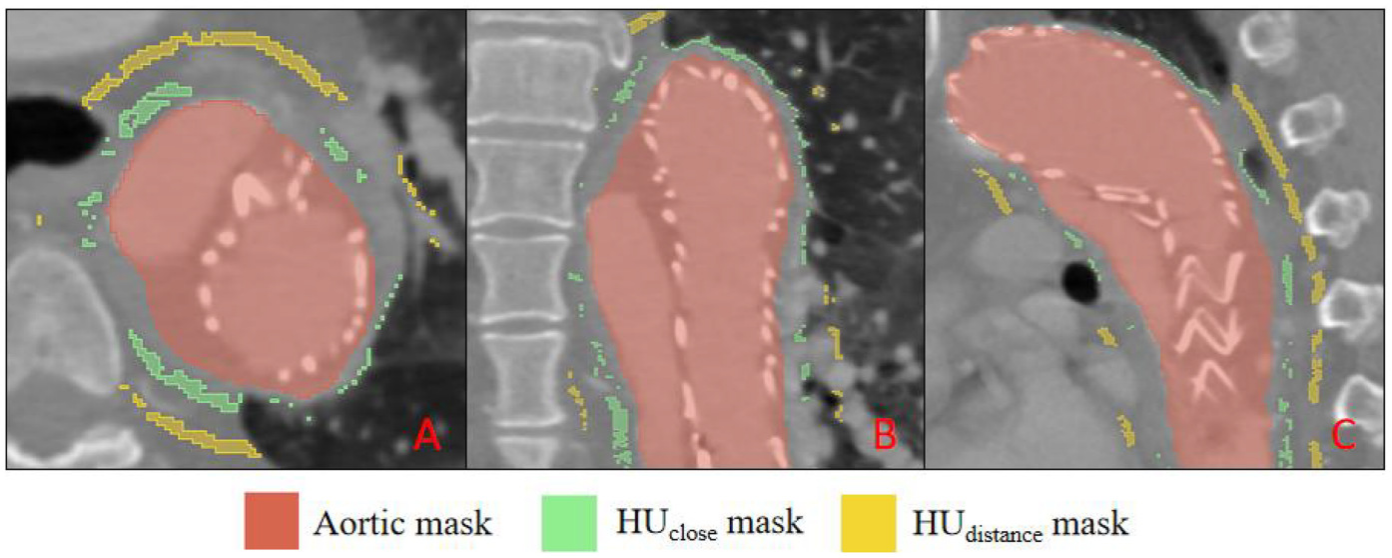
Multiplanar CT segmentation of perivascular adipose tissue (PVAT). **(A)** shows an axial (transverse) slice, **(B)** a coronal slice, and C a sagittal slice. The aortic lumen is delineated in red (aortic mask); PVAT immediately adjacent to the aortic wall is highlighted in green (HU close mask), while PVAT located farther from the wall is indicated in yellow (HU distance mask).

Two quantitative indices were derived: HU_Δ_ = HU_close_ −HU_distant_ and HU_ratio_ = HU_close_/HU_distant_ (17). The resulting PVAT masks are illustrated in Figure 4. Three-dimensional centreline extraction and visualisation of aortic dissection were performed with 3D Slicer and CREALIFE software (http://www.crealifemed.com) (22).

### Statistical analysis

Statistical analyses were conducted with Python 3.12 (pandas 2.2, SciPy 1.13, statsmodels 0.15). Baseline characteristics were compared between the two groups. Normality of continuous variables was evaluated with the Shapiro–Wilk test; data conforming to a normal distribution were analysed with the independent-samples *t*-test, whereas non-normal data were compared with the Wilcoxon rank-sum test. Categorical variables were assessed using the *χ*² test. All tests were two-sided and a *P* < 0.05 denoted statistical significance. Patients in whom either the proximal or distal PVAT thickness was <0.3 cm were excluded to avoid measurement error.

Subsequently, univariate logistic regression was performed for each candidate predictor, with results reported as odds ratios (OR), 95% confidence intervals (CI) and *P*-values. Predictors with *P* < 0.05 advanced to multivariate Logistics modelling, where collinearity was assessed via the variance-inflation factor (VIF); variables with VIF >10 were removed or combined. Finally, receiver-operating characteristic (ROC) analysis was applied to identify the optimal cut-off for predicting a residual false lumen after TEVAR, using Youden's index to maximise sensitivity and specificity.

### HU visualization of PVAT

**Figure 5:**
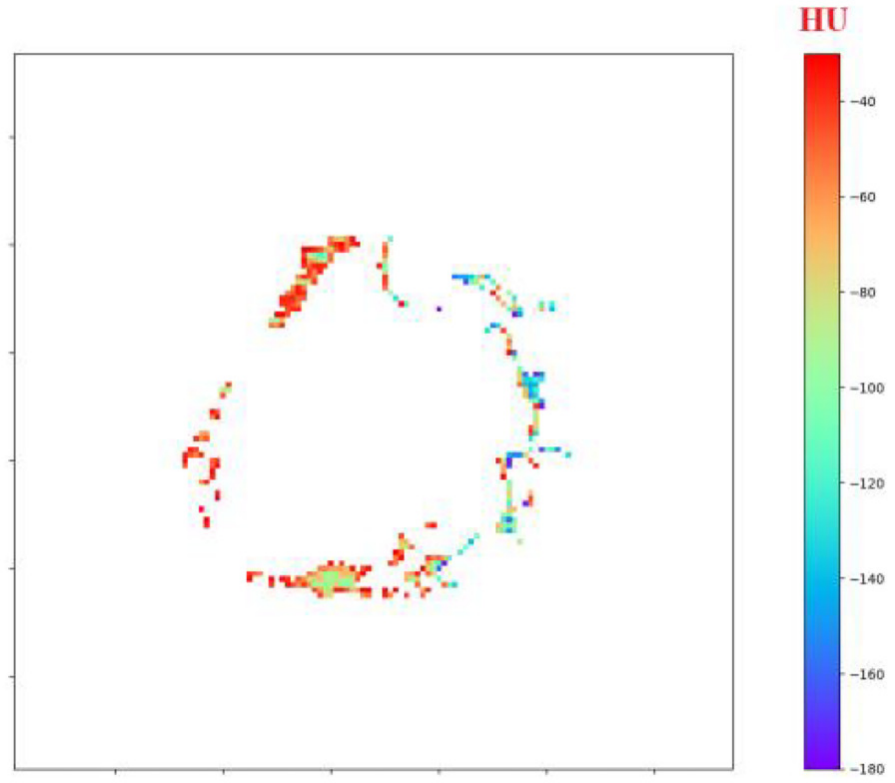
Interactive visualization of perivascular adipose tissue (PVAT) surrounding the stented aorta.

Using the custom Python tool built on SimpleITK 2.3, NumPy and Matplotlib 3.9, the adipose mask voxels filtered between −180 HU and −30 HU are rendered slice-by-slice with a perceptually uniform rainbow colour-map. An interactive slider scrolls through the axial volume, a colour-bar anchors the HU scale, and the title line updates in real time with the mean attenuation of the displayed slice, giving an at-a-glance view of regional PVAT heterogeneity around the stent, as shown in Figure 5.

## Result

### Baseline comparison

Among the 132 patients, 56 (42.4%) were classified into Group 1 (residual false lumen) and 76 (57.6%) into Group 2 (closed false lumen); demographic variables—age, BMI, sex distribution, smoking and drinking status—and comorbid conditions such as diabetes and hypertension were comparable between the two groups (all *P* > 0.05); postoperative laboratory tests revealed a higher white-blood-cell count in Group 1 (15.27 ± 3.76 × 10⁹/L vs. 13.69 ± 4.28 × 10⁹/L, *P* = 0.026) together with a shorter activated partial-thromboplastin time (40.10 ± 10.60 s vs. 44.12 ± 11.44 s, *P* = 0.039) and lower D-dimer levels (1,262 ± 643 ng/L vs. 1,502 ± 639 ng/L, *P* = 0.036), whereas lipid profiles and other hematologic indices did not differ significantly; regarding imaging data, Group 1 exhibited a larger HU_Δ_ (8.75 ± 3.29 vs. 5.16 ± 2.84, *P* < 0.001) and a smaller HU_ratio_ (0.73 ± 0.13 vs. 0.85 ± 0.11, *P* < 0.001); with respect to TEVAR procedural variables, the interval from admission to surgery (3.96 ± 2.10 d vs. 4.44 ± 2.28 d, *P* = 0.216) and graft oversizing rate (10.41 ± 2.13% vs. 9.70 ± 3.18%, *P* = 0.126) were similar, but Group 2 received a longer stent relative to tear length (86.43 ± 8.87% vs. 82.43 ± 8.40%, *P* = 0.009), and the choice of graft covering material (ePTFE vs. Dacron) did not differ significantly between the cohorts *(P* = 0.216), as shown in Table 1.

**Table 1:** Demographic characteristics of the two groups.

Variable	Overall (*n* = 132)	Group 1 (*n* = 56)	Group 2 (*n* = 76)	*P-value*
Demographic data				
Age (years)	62.38 ± 11.71	64.64 ± 11.15	60.71 ± 11.90	0.054
BMI (kg/m²)	22.97 ± 2.47	22.79 ± 2.35	23.10 ± 2.56	0.462
Gender (*n*, %)				0.319
Female	32 (24.24%)	16 (28.57%)	16 (21.05%)	
Male	100 (75.76%)	40 (71.43%)	60 (78.95%)	
Smoke (*n*, %)				0.684
Non	99 (75%)	43 (76.79%)	56 (73.68%)	
Yes	33 (25%)	13 (23.21%)	20 (26.32%)	
Drink (*n*, %)				0.146
Non	108 (81.82%)	49 (87.50%)	59 (77.63%)	
Yes	24 (18.18%)	7 (12.50%)	17 (22.37%)	
Length of Stay (day)	13.50 ± 3.93	13.50 ± 3.85	13.50 ± 4.07	0.999
Complication				
Diabetes (*n*, %)				0.267
Non	118 (89.39%)	52 (92.86%)	66 (86.84%)	
Yes	14 (10.61%)	4 (7.14%)	10 (13.16%)	
Hypertension (*n*, %)				0.326
Non	56 (42.42%)	21 (37.50%)	35 (46.05%)	
Yes	76 (57.58%)	35 (62.50%)	41 (53.95%)	
Blood biochemistry data				
WBC(×10⁹/L)	14.36 ± 4.12	15.27 ± 3.76	13.69 ± 4.28	0.026
Neutrophil count(×10⁹/L)	9.87 ± 3.71	10.59 ± 3.59	9.35 ± 3.73	0.056
Lymphocyte count(×10⁹/L)	1.88 ± 0.94	1.95 ± 0.85	1.83 ± 1.01	0.457
LDL(mg/dl)	126.74 ± 22.28	129.36 ± 24.92	124.81 ± 20.07	0.263
HDL(mg/dl)	37.71 ± 15.57	40.60 ± 14.99	35.58 ± 15.75	0.065
TC(mg/dl)	202.79 ± 18.97	203.79 ± 20.21	202.05 ± 18.11	0.610
PT(s)	18.18 ± 6.08	18.53 ± 4.70	17.93 ± 6.94	0.554
APTT(s)	42.42 ± 11.23	40.10 ± 10.60	44.12 ± 11.44	0.039
D-Dimer(ng/L)	1,400.47 ± 649.28	1,262.28 ± 643.65	1,502.30 ± 638.56	0.036
Image features				
HU_ratio_	0.80 ± 0.13	0.73 ± 0.13	0.85 ± 0.11	<0.001
HU_Δ_	6.68 ± 3.51	8.75 ± 3.29	5.16 ± 2.84	<0.001
TEVAR surgical data				
Surgery time(day)	4.23 ± 2.21	3.96 ± 2.10	4.44 ± 2.28	0.216
Oversizing rate (%)	10.00 ± 2.80	10.41 ± 2.13	9.70 ± 3.18	0.126
Stent length/tear length × 100% (%)	84.73 ± 8.87	82.43 ± 8.40	86.43 ± 8.87	0.009
Stent graft membrane material				0.216
Dacron (*n*, %)	98 (74.24%)	60 (78.95%)	38 (67.86%)	
ePTFE (*n*, %)	34 (25.76%)	16 (21.05%)	18 (32.14%)	

### Logistic regression and predictors

Univariate logistic regression confirmed significant associations between residual false lumen and WBC count, APTT, D-dimer, HUΔ, HUratio, and the stent-to-tear length ratio (all *P* < 0.05); after verifying that multicollinearity was acceptable for these candidates (VIF < 10), a multivariate model showed that only the two CT attenuation metrics remained independently predictive—each unit decrease in HUratio markedly reduced the odds of a residual false lumen (*β* = –9.01, 95% CI −13.796 to −4.876, *P* < 0.001), while each unit increase in HUΔ increased the odds (*β* = 0.343, 95% CI 0.183–0.530, *P* < 0.001); inflammatory (WBC) and coagulation markers (APTT, D-dimer), together with the stent-coverage variable, lost statistical significance after adjustment, suggesting their apparent effects were largely mediated through the imaging-derived parameters, as shown in Table 2.

**Table 2:** Univariate and multivariate modeling for prediction of distal endoleak after TEVAR.

Variable	Univariate analysis		Multivariate analysis	
	95% confidence interval (CI)	*P-value*	95% confidence interval (CI)	*P-value*
Demographic data				
Age (years)	(−0.001, 0.061)	0.059		
Gender	(−1.206, 0.395)	0.321		
BMI(kg/m²)	(−0.194, 0.089)	0.465		
Smoke	(−0.97, 0.637)	0.684		
Drink	(−1.66, 0.257)	0.151		
Complication				
Diabetes	(−1.893, 0.537)	0.274		
Hypertension	(−0.352, 1.057)	0.327		
Blood biochemistry data				
WBC(×10⁹/L)	(0.009, 0.185)	0.031	(−0.076, 0.160)	0.480
Neutrophil count (×10⁹/L)	(−0.004, 0.192)	0.06		
Lymphocyte count (×10⁹/L)	(−0.232, 0.507)	0.466		
LDL(mg/dl)	(−0.006, 0.025)	0.247		
HDL(mg/dl)	(−0.002, 0.044)	0.069		
TC(mg/dl)	(−0.013, 0.023)	0.601		
PT(s)	(−0.041, 0.074)	0.573		
APTT(s)	(−0.065, −0.001)	0.044	(−0.062, 0.026)	0.435
D-Dimer(ng/L)	(−0.001, 0.000)	0.038	(−0.002, 0.001)	0.068
Image features				
HU_Δ_	(0.239, 0.540)	<0.001	(0.183, 0.530)	<0.001
HU_ratio_	(−12.533, −5.395)	<0.001	(−13.796, −4.876)	<0.001
TEVAR surgical data				
Surgery time(day)	(−0.264, 0.058)	0.766		
Oversizing rate (%)	(−3.167, 22.647)	0.151		
Stent length/tear length × 100%(%)	(−9.618, −1.264)	0.012	(−10.382, 0.106)	0.058
Stent graft membrane material	(−1.370, 0.211)	0.152		

### ROC analysis

**Figure 6:**
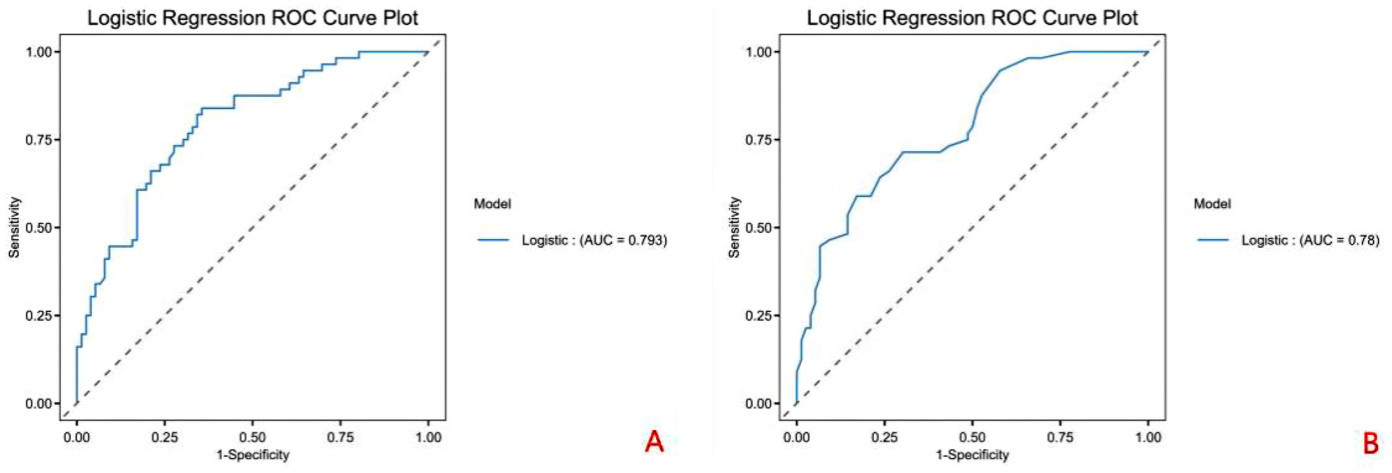
ROC curve analysis. **(A)** ROC curve for HU Δ; **(B)** ROC curve for HU ratio.

ROC curve analysis identified optimal cut-off values for predicting residual false lumen: HU_Δ_ > 7.170 and HU_ratio_ ≤ 0.790, with an AUC of 0.79, as shown in Figure 6.

All continuous variables are reported as mean ± standard deviation and categorical variables as count (percentage); white-blood-cell count (WBC), neutrophil count, lymphocyte count, prothrombin time (PT), activated partial thromboplastin time (APTT) and D-dimer were obtained from the first postoperative laboratory panel after thoracic endovascular aortic repair (TEVAR), whereas low-density lipoprotein cholesterol (LDL), high-density lipoprotein cholesterol (HDL) and total cholesterol (TC) were measured on admission; imaging metrics include HUratio—the ratio of mean arterial-phase CT Hounsfield-unit attenuation in the false lumen to that in the true lumen—and HUΔ, the absolute HU difference between those two measurements; in the TEVAR section, surgery time (day) represents the interval in days from diagnosis to completion of the procedure, oversizing rate (%) = (nominal stent-graft diameter—native aortic diameter)/(native aortic diameter) × 100%, and Stent length/tear length × 100% denotes the percentage of stent length relative to the length of the primary intimal tear; stent-graft membrane material is classified as expanded polytetrafluoroethylene (ePTFE) or Dacron; length of stay (day) is defined as the total duration from hospital admission to discharge; comparisons between Group 1 and Group 2 were performed with appropriate statistical tests, and *P* < 0.05 was considered statistically significant; abbreviations: APTT, activated partial thromboplastin time; BMI, body-mass index; D-dimer, fibrin degradation product; ePTFE, expanded polytetrafluoroethylene; HDL, high-density lipoprotein cholesterol; HU, Hounsfield unit; LDL, low-density lipoprotein cholesterol; PT, prothrombin time; TC, total cholesterol; TEVAR, thoracic endovascular aortic repair; WBC, white-blood-cell count.

All continuous variables are reported as mean ± standard deviation and categorical variables as count (percentage); white-blood-cell count (WBC), neutrophil count, lymphocyte count, prothrombin time (PT), activated partial thromboplastin time (APTT) and D-dimer were obtained from the first postoperative laboratory panel after thoracic endovascular aortic repair (TEVAR), whereas low-density lipoprotein cholesterol (LDL), high-density lipoprotein cholesterol (HDL) and total cholesterol (TC) were measured on admission; imaging metrics include HUratio—the ratio of mean arterial-phase CT Hounsfield-unit attenuation in the false lumen to that in the true lumen—and HUΔ, the absolute HU difference between those two measurements; in the TEVAR section, surgery time (day) represents the interval in days from diagnosis to completion of the procedure, oversizing rate (%) = (nominal stent-graft diameter—native aortic diameter)/(native aortic diameter) × 100%, and Stent length/tear length × 100% denotes the percentage of stent length relative to the length of the primary intimal tear; stent-graft membrane material is classified as expanded polytetrafluoroethylene (ePTFE) or Dacron; length of stay (day) is defined as the total duration from hospital admission to discharge; comparisons between Group 1 and Group 2 were performed with appropriate statistical tests, and *P* < 0.05 was considered statistically significant; abbreviations: APTT, activated partial thromboplastin time; BMI, body-mass index; D-dimer, fibrin degradation product; ePTFE, expanded polytetrafluoroethylene; HDL, high-density lipoprotein cholesterol; HU, Hounsfield unit; LDL, low-density lipoprotein cholesterol; PT, prothrombin time; TC, total cholesterol; TEVAR, thoracic endovascular aortic repair; WBC, white-blood-cell count.

## Discussion

In this study, we explore the role of PVAT in the development of residual false lumen formation following TEVAR in patients with TBAD. Our findings indicate that the attenuation of PVAT, particularly the difference in density between regions near and far from the aortic wall (HU*_Δ_*) and the ratio of these values (HU_ratio_), are significant predictors of residual false lumen formation post-TEVAR. These results suggest that PVAT could serve as a valuable non-invasive biomarker for predicting postoperative vascular outcomes and identifying high-risk patients. The association between PVAT and residual false lumen formation underscores the potential of PVAT as a key indicator of the vascular environment following TEVAR and highlights the need for further investigation into its underlying pathophysiological mechanisms.

The basic principle of TEVAR is to deploy a covered stent within the true lumen of the aortic dissection, which expands the true lumen and reduces the false lumen by promoting thrombosis (23). This reduces the risk of aortic rupture by sealing the proximal rupture site. With advances in TEVAR technology, postoperative complications such as endoleaks, stroke, and organ ischemia have been significantly reduced (24, 25). However, challenges remain, particularly the issue of residual false lumen formation at the distal end of the stent. This complication arises because the stent generally seals only the proximal rupture, leaving the distal decompression port uncovered. As a result, residual channels remain between the true and false lumens. While most of these residual false lumens undergo thrombosis over time, some may progress to form aneurysms, which carry the risk of rupture and potentially fatal outcomes (26, 27). Identifying high-risk patients with residual false lumen in the early postoperative period and providing timely intervention could significantly improve treatment outcomes and long-term prognosis.

Previous studies have primarily focused on factors such as the geometric structure of the aorta, inflammatory markers, platelet activation markers, coagulation factors like D-dimer, coated stent material and hemodynamics (28, 29). These factors are all closely related to the structural remodeling of the aortic dissection and are implicated in its occurrence, progression, treatment, and prognosis. Recently, there has been increasing recognition of the critical role of perivascular adipose tissue in the development of vascular diseases such as coronary artery disease and aortic aneurysm (17, 18). PVAT, a specialized type of adipose tissue, surrounds most blood vessels, excluding the cerebral vasculature (30). This metabolically active tissue consists of both white and brown adipose tissue (31). White adipose tissue serves as an energy reservoir and secretes various hormones, cytokines, and enzymes that influence inflammation, metabolism, and vascular homeostasis. Brown adipose tissue is thought to contribute to thermogenesis (32, 33). Studies have shown that the thoracic aorta is primarily composed of brown adipose tissue, while the abdominal aorta is mainly composed of white adipose tissue (34, 35). Under normal physiological conditions, PVAT is involved in the regulation of vascular tone. However, under pathological conditions, PVAT can exacerbate oxidative stress, leading to adverse vascular remodeling. Although studies have established the role of PVAT in the development and prognosis of abdominal aortic aneurysms following endovascular repair, its role in false lumen remodeling after TEVAR remains underexplored (36). This study aims to address this gap by investigating the correlation between PVAT and residual false lumen formation post-TEVAR.

We retrospectively analyzed a cohort of 132 patients with TBAD who underwent TEVAR at our institution. We collected baseline data, biochemical indicators, and imaging data. The patients were classified into two groups: those with residual false lumen and those with closed false lumen, based on the status of the aortic dissection around the stent at discharge. There were no significant differences in baseline characteristics such as age, gender, BMI, comorbidities, or blood lipid levels between the two groups, ensuring comparability. Comparison of early postoperative blood tests revealed that white blood cell count (WBC), activated partial thromboplastin time (APTT), and D-dimer levels were significantly higher in the residual false lumen group compared to the closed false lumen group. This finding is consistent with the work of Kimura et al., which emphasized the impact of inflammatory factors on the long-term outcome of the false lumen following TEVAR (37). This study further corroborates that early postoperative coagulation parameters, such as D-dimer and APTT, significantly influence residual false lumen formation and should be closely monitored during the early postoperative period.

Although our multivariate analysis identified HU_Δ_ and HU_ratio_ as independent predictors, several unmeasured or incompletely adjusted variables may confound the observed associations. First, BMI influences both the quantity and metabolic phenotype of PVAT; obesity-induced whitening of brown PVAT can lead to lower CT attenuation values regardless of dissection status (31, 32). Second, the widespread perioperative use of statins and other lipid-lowering or anti-inflammatory agents in TBAD patients may reduce PVAT density through pleiotropic effects, potentially attenuating HU_Δ_ and HU_ratio_ and blunting their predictive accuracy. Third, systemic inflammatory states—ranging from postoperative systemic inflammatory response syndrome to chronic low-grade inflammation associated with metabolic syndrome—could simultaneously increase PVAT attenuation and impair false-lumen thrombosis, thereby acting as a mediating pathway. Propensity-matched or longitudinal designs that explicitly control for these variables will be essential for isolating the true causal contribution of PVAT characteristics to false-lumen persistence.

Our quantitative analysis demonstrated that patients with residual false lumen had significantly higher HU_Δ_ values and lower HU_ratio_ values compared to those with closed false lumen (*P* < 0.001 for both). Moreover, logistic regression confirmed that early postoperative HU_Δ_ and HUratio are independent risk factors for residual false lumen formation around the stent in the short term after TEVAR. ROC curve analysis indicated that the optimal threshold for HU_Δ_ was 7.170, with a sensitivity of 0.895 and a specificity of 0.762, while the optimal threshold for HUratio was 0.790, with a sensitivity of 0.947 and a specificity of 0.667.

Translating these findings into practice, HU_Δ_ and HU_ratio_ can be incorporated into a tiered surveillance algorithm. Patients exceeding the high-risk cut-offs (> 7.170 for HU_Δ_ or ≤ 0.790 for HU_ratio_) could undergo an intensified imaging schedule—CTA or MRA at 1–3 months, followed by 6-monthly scans during the first year—while those below the thresholds continue with standard follow-up. Likewise, knowledge of an adverse PVAT profile pre-operatively or on immediate postoperative CTA may encourage more aggressive intra-operative strategies, such as extended-coverage stent grafting, adjunctive distal bare-metal stenting, or prophylactic left subclavian revascularization (23, 28). From a medical-management standpoint, early initiation or up-titration of statins, β-blockers, ACE inhibitors, and anti-inflammatory agents might help modulate PVAT activity and foster false-lumen thrombosis (38–40). Finally, serial assessment of PVAT attenuation during routine scans provides a dynamic biomarker that could trigger timely re-intervention—endovascular fenestration, coil embolization, or open conversion—before aneurysmal degeneration ensues.

Previous research has shown that HU*_Δ_* and HU_ratio_ values reflect the degree of vascular wall inflammation by quantifying the attenuation of adipose tissue around the aortic wall (41). Our study further elucidates that greater attenuation may correlate with more severe inflammation in the vascular wall, contributing to adverse remodeling of the false lumen following TEVAR. In addition, HU*_Δ_* and HU_ratio_ values may also be related to local coagulation changes within the vessel, suggesting a complex interplay between inflammation and coagulation that warrants further investigation. These findings provide valuable insights into the pathophysiological mechanisms underlying residual false lumen formation and underscore the potential of PVAT as a non-invasive biomarker for predicting postoperative outcomes in TEVAR-treated patients.

Looking ahead, targeting PVAT represents a promising supplementary treatment approach following TEVAR (42). Given its significant role in vascular inflammation and remodeling, modulating PVAT could help reduce the risk of residual false lumen formation post-surgery. Therapeutic strategies aimed at reducing oxidative stress, modulating the release of inflammatory cytokines, or enhancing PVAT's regenerative potential could be integrated into TEVAR treatment regimens. By targeting PVAT, clinicians may be able to further improve postoperative vascular health and reduce long-term complications such as aneurysm formation or rupture. However, further research is needed to explore the safety, feasibility, and efficacy of PVAT-targeted therapies as adjunctive treatments in TEVAR. The integration of such strategies could provide a significant improvement in the long-term outcomes for patients undergoing TEVAR.

## Limitation

One limitation of this study is its retrospective design, which may introduce selection bias and limit the generalizability of the findings. The sample size, while adequate, is relatively small, and larger, multicenter studies are needed to validate the results. Additionally, while we focused on the early postoperative period, the long-term effects of PVAT attenuation on false lumen remodeling were not assessed. Another limitation is the reliance on imaging techniques for measuring PVAT density, which, despite advances in technology, may still be subject to variability in interpretation and resolution. Finally, although the TotalSegmenter model provides a novel approach for extracting PVAT density values, it has not been extensively validated in a broader clinical context, and further refinement and testing of this tool are required to ensure its widespread applicability.

## Conclusion

In conclusion, this study highlights the significant role of perivascular adipose tissue (PVAT) attenuation, particularly HU_Δ_ and HU_ratio_, as key predictors of residual false lumen formation following TEVAR in patients with Stanford Type B aortic dissection. PVAT attenuation may serve as a valuable non-invasive biomarker for identifying high-risk patients and predicting postoperative outcomes. These findings underscore the potential of PVAT in guiding clinical decision-making and highlight the need for further research into PVAT's underlying mechanisms and its potential role in adjunctive therapies for improving long-term TEVAR outcomes.

## Data Availability

The raw data supporting the conclusions of this article will be made available by the authors, without undue reservation.
